# Risk Factors for In-Hospital Mortality from COVID-19 Among Nursing Home Patients—An Urban Center Experience

**DOI:** 10.1017/ash.2021.90

**Published:** 2021-07-29

**Authors:** Amit Vahia, Mamta Sharma, Leonard Johnson, Ashish Bhargava, Louis Saravolatz, Susan Szpunar

## Abstract

**Background:** As the COVID-19 pandemic continues, special attention is focused on high-risk patients. In this study, we assessed the risk factors for COVID-19 mortality in nursing home patients. **Methods:** In this retrospective cohort study, we reviewed the electronic medical records of SARS-COV-2 PCR–positive nursing-home patients between March 8 and June 14, 2020. The primary outcome was in-hospital mortality. Risk factors were compared between those who were discharged or died using the Student *t* test, the Mann-Whitney U test, χ^2^ analysis, and logistic regression. **Results:** Among 169 hospitalized nursing-home patients, the case fatality rate was 43.2%. The mean age was 72.3 ± 13.8 years; 92 patients (54.4%) were male; and 112 patients (66.3%) were black. Within the first day of hospitalization, 83 (49%) patients developed fever. On admission, 24 (14.2%) patients were hypotensive. Leukopenia, lymphopenia, and thrombocytopenia were present in 20 (12%), 91 (53%), and 40 (23.7%) patients, respectively. Among the inflammatory markers, elevations in CRP and ferritin levels occurred in 79% and 24%, respectively. Intensive care admission was needed for 40 patients (23.7%). Septic shock occurred in 25 patients (14.8%). Patients over the age of 70 were more likely to die than younger patients (OR, 2.2; 95% CI, 1.2– 4.1; *P* = .20). Patients with a fever on admission were more likely to die than those who were afebrile (OR, 2.03; 95% CI, 1.08–3.8; *P* = .03). Also, 66.7% hypotensive patients died compared to 39.3% normotensive patients (OR, 3.1; 95% CI, 1.2–7.7 *P* = .01). Intubated patients died more often than those not intubated, 78.4% versus 33.3%, respectively (OR=7.3, p < 0.001, CI 3.1, 17.2) Factors significantly associated with death included higher mean qSofa (p < 0.001), higher median Charlson scores (0.02), thrombocytopenia (p = 0.04) and lymphocytopenia (0.04). From multivariable logistic regression, independent factors associated with death were Charlson score (OR=1.2, p=0.05), qSofa (OR=2.0, p=0.004), thrombocytopenia (OR = 3.0, p = 0.01) and BMI less than 25 (OR = 3.5, p=0.002). **Conclusions:** Our multivariable analysis revealed that patients with a greater burden of comorbidities, lower BMI, higher qSOFA sepsis score, and thrombocytopenia had a higher risk of death, perhaps because of severe infection despite a robust immune response.

**Funding:** No

**Disclosures:** None

